# ﻿Selaginellawuyishanensis (sect. ﻿Tetragonostachyae, Selaginellaceae), a new species from East China and its phylogenetic position based on molecular data

**DOI:** 10.3897/phytokeys.202.85410

**Published:** 2022-07-28

**Authors:** Ke-Wang Xu, Shui-Fei Chen, Qiang Song, Xiao Zheng, Meng Li, Yan-Ming Fang, Hong-Jin Wei, Hui Ding, Xin-Mao Zhou, Yi-Fan Duan

**Affiliations:** 1 Co-Innovation Center for Sustainable Forestry in Southern China & Key Laboratory of National Forestry and Grassland Administration on Subtropical Forest Biodiversity Conservation, College of Biology and the Environment, Nanjing Forestry University, Nanjing, 210037, China Nanjing Forestry University Nanjing China; 2 Nanjing institute of Environmental Sciences, State Environmental Protection Scientific Observation and Research Station for Ecological Environment of Wuyi Mountains, State Environmental Protection Key Laboratory on Biosafety, Ministry of Ecology and Environment, Nanjing 210037, China Nanjing institute of Environmental Sciences Nanjing China; 3 Shanghai Chenshan Botanical Garden, Shanghai, 201602, China Shanghai Chenshan Botanical Garden Shanghai China; 4 School of Ecology and Environmental Science, Yunnan University, Kunming, 650091, Yunnan, China Yunnan University Kunming China

**Keywords:** Danxia landform, *Selaginellaalbociliata*, *Selaginella* subg. *Heterostachys*, species diversity

## Abstract

A new spikemoss species, *Selaginellawuyishanensis*, is described and illustrated based on materials collected from Fujian Province, East China. The new species can be distinguished from *S.lutchuensis* Koidzumi and *S.albociliata* P. S. Wang by its leaves with extremely long cilia (up to 8 mm) and distinctly white margins, ovate ventral sporophylls, and sporophyll-pteryx completely inverted on dorsal sporophylls. In the present work, a molecular phylogeny, taxonomic description, distribution information, line drawing, and photographs of this new species are presented. A morphological comparison is also given to distinguish it from morphologically similar species in Selaginellasect.Tetragonostachyae (Hook. & Grev.) Hieron. & Sadeb.

## ﻿Introduction

*Selaginella* P. Beauv. (Selaginellaceae) is the largest genus of seed-free vascular plants with more than 700 species worldwide (Jermy 1990; Zhou and Zhang 2015; PPG 2016; Weststrand and Korall 2016a). It is mainly distributed in tropical and subtropical regions, with a few species reaching the arctic-alpine zones in both hemispheres (Jermy 1990; Zhang et al. 2013). Members of *Selaginella* are heterosporous, and usually have rhizophores, leaves arranged in four rows, and terminal strobili. Though the subgeneric classification of *Selaginella* has been controversial (Zhou and Zhang 2015; Weststrand and Korall 2016b), integrative taxonomy based on morphological, cytological, and molecular data can effectively provide new insights into the species delimitation and discovery of new taxa (Zhou and Zhang 2015; Zhou et al. 2015a, b, 2016; Wu et al. 2017; Ye et al. 2020; Zhang et al. 2021; Wang et al. 2022).

Since 2019, we have conducted investigations into wild vascular plants in Wuyishan National Nature Reserve (**WNNR**) in Fujian Province, East China. Most of the *Selaginella* species we have encountered are common in WNNR and can be easily identified to described species. However, one species collected from Danxia regions of WNNR didn’t match species listed in available checklists and monographs or those recently described species from East Asia (Zhang et al. 2013; Zhou et al. 2015a, b; Wu et al. 2017; Shalimov et al. 2019; Ye et al. 2020; Zhang et al. 2021; Wang et al. 2022). Based on morphological study of herbarium specimens and consultation of literature, we found that this species is most similar to *S.albociliata*, but this species has smooth megaspore surfaces and ovate-lanceolate ventral sporophylls. In order to infer the phylogenetic relationships of this species, we conducted a phylogenetic analysis using both plastid and nuclear loci. With evidence from morphological characters and molecular phylogeny, we propose it as a new spikemoss species, and describe and illustrate it herein.

## ﻿Materials and methods

The gross morphology of the new species was observed and examined both from the fresh plants and dried herbarium specimens using SMZ1270 stereomicroscope (Nikon, Japan). For spore morphology, a scanning electron microscope (SEM) was used to observe the megaspores and microspores. Spore samples obtained from herbarium specimen were mounted on specimen tabs and then coated with platinum in a sputter coater. Observations were conducted using an ESEM-Quanta 200 (FEI, Hillsboro, Oregon, USA) with 15 Kv at Nanjing Forestry University, Nanjing, China. The quantitative characters of the new species were measured using the ImageJ software (Pérez and Pascau 2013). Voucher specimens (see Appendix 1) were deposited at NF and PYU (herbaria acronyms according to Thiers 2018).

For the phylogenetic study, a total of 84 accessions representing 50 species of the genus were included, of which four accessions representing four species were selected as outgroups based on earlier phylogenetic analysis (Zhou et al. 2016; Weststrand and Korall 2016b). Three samples from three different populations of the new species were newly sequenced. Total genomic DNA was extracted from silica-dried leaves using a TIANGEN plant genomic DNA extraction kit (TIANGEN Biotech, Beijing, China) Mini Kits (Qiagen, Germany) following the manufacturer’s protocols. One plastid gene *rbcL* and one nuclear region *ITS* were selected for the phylogenetic analysis based on Zhou et al. (2016). The PCR and sequencing protocols follow Zhou et al. (2016). The newly generated sequences were assembled and edited using Sequencher ver. 4.14 (GeneCodes Corporation, Ann Arbor, Michigan). All sequences of *rbcL* and ITS regions were initially aligned with MAFFT ver. 7 (Katoh and Standley 2013) and manually adjusted in BioEdit (Hall 1999). The two alignments were concatenated and the final combined dataset was analyzed with maximum likelihood (ML) and Bayesian inference (BI) methods. The ML tree searches were performed using RAxML-HPC2 on XSEDE with 1000 bootstrap replicates. The model GTR+I+G was chosen for the combined dataset using the AIC criterion with JModelTest 2 (Darriba et al. 2012). The BI was conducted using MrBayes ver. 3.2.7a (Ronquist and Huelsenbeck 2003) with temperature parameter set to 0.2, and keeping other parameters consistent with the default parameters of the software. Two independent runs of four Markov chain Monte Carlo chains, each with four chains (one cold, three heated), were conducted, each beginning with a random tree and sampling one tree every 1000 generations of 10 000 000 generations. Convergence among runs and stationarity were assessed using Tracer ver. 1.4 (Rambaut and Drummond 2007), and the first 25% was discarded as burnin. The remaining trees were used to calculate a 50% majority-rule consensus topology and posterior probabilities (PP).

## ﻿Results and discussion

**Figure 1.**
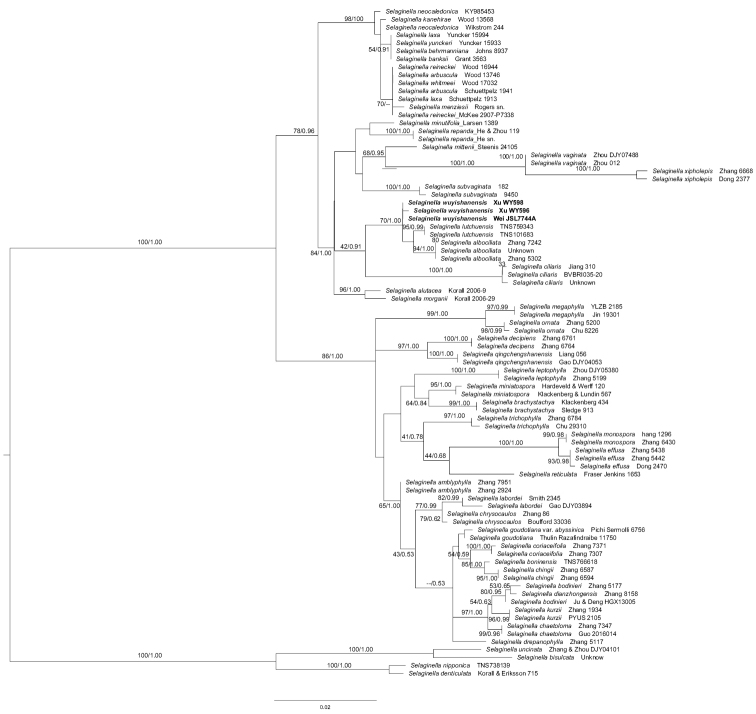
The maximum likelihood phylogeny of *Selaginellawuyishanensis* and its congeners based on plastid gene *rbcL* and nuclear locus ITS. Support values [maximum likelihood bootstrap support (MLBS) ≥ 50%, Bayesian inference posterior probability (BIPP) ≥ 0.5] are shown above the main branches. The dash (--) indicates MLBS < 50% or BIPP < 0.5.

Based on our phylogenetic analysis, three collections from three different populations of the new species is in a polytomy with *S.lutchensis* and *S.albociliata* (Fig. 1). Three samples of this new species are not resolved as a monophyletic group. However, both *S.lutchuensis* and *S.albociliata* are monophyletic and they form a strongly supported clade together (MLBS = 95% and BIPP = 0.99) (Fig. 1). Morphologically, all the three species have leaves ciliate along the margin. Based on the recent infrageneric classification of *Selaginella* proposed by Zhou and Zhang (2015), *S.wuyishanensis* should be assigned to S.subg.HeterostachysBakersect.Tetragonostachyae (Hook. & Grev.) Hieron. & Sadeb.

**Figure 2.**
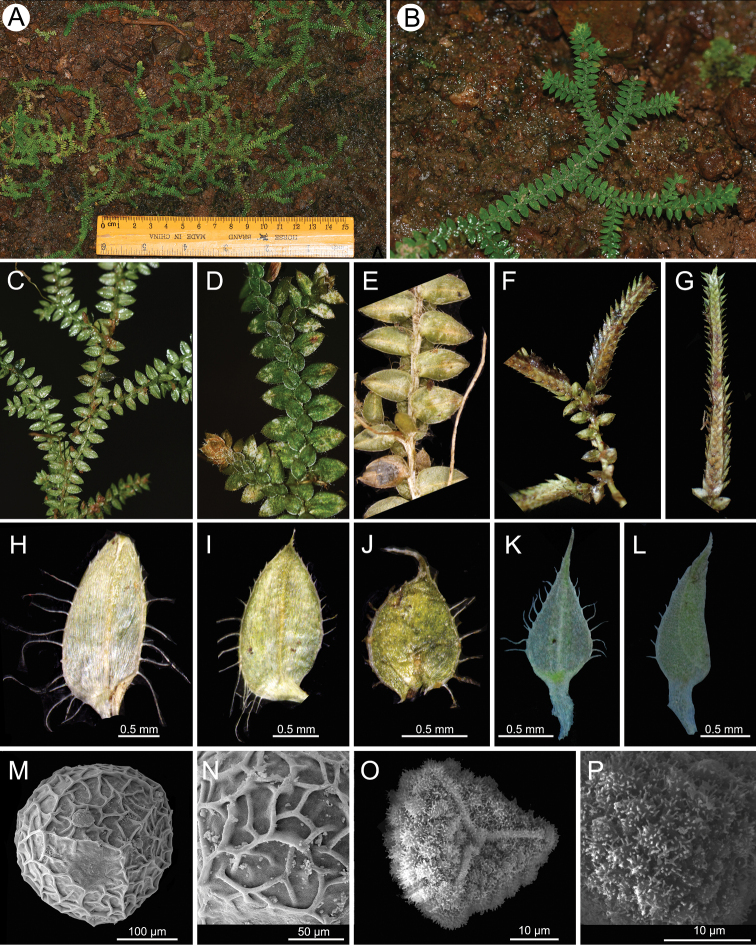
*Selaginellawuyishanensis***A, B** habit **C** abaxial view of portion of branch **D** portion of branch showing the dorsal leaves **E** portion of branch showing the ventral and axillary leaves **F, G** strobili **H** axillary leave **I** axillary leave **J** dorsal leave **K** ventral sporophyll **L** dorsal sporophyll **M** proximal surface of megaspores **N** detail of megaspore surface **O** microscopic structures of microspore surface **P** proximal surface of microspore.

Morphologically, *Selaginellawuyishanensis* is most similar to *S.albociliata* and *S.lutchuensis* in having plants creeping (Fig. 2A), leaf margins white (Fig. 2H–L), axillary and ventral leaves ciliate along the margins (Fig. 2H–L), megaspore surfaces reticulate ornamentation with fine muri (Fig. 2M, N), and microspore surfaces verrucate (Fig. 2O, P), but *S.wuyishanensis* has long cilia on ventral leaves up to 0.6 mm long (Fig. 2H; vs. short cilia up to 0.2 mm long in *S.lutchuensis*), dorsal leaves long ciliate (Fig. 2J; vs. loosely serrulate in *S.lutchuensis*), verrucate ornamentation covered with small rodlet in microspore (Fig. 2P; vs. covered with coral-like structures in *S.lutchuensis*: Chang et al. 2009). *Selaginellawuyishanensis* is morphologically also similar to *S.albociliata* in having plants epilithic and leaves ciliate along the margins. However, *S.wuyishanensis* has smooth megaspore surfaces (Fig. 2M, N; vs. megaspore surface with fine and low papillae structure in *S.albociliata*: Zhou et al. 2015b), ventral sporophylls ovate with length-to-width ratio of ca. 2.4 (Fig. 2K; vs. ovate-lanceolate with length-to-width ratio of ca. 3.2 in *S.albociliata*). In geography, *S.wuyishanensis* is only known to occur in the Danxia landform of East China, whereas *S.albociliata* is restricted to the limestone mountains of the karst regions in the Southwest (Guizhou) and South (Guangxi) China.

### ﻿Taxonomic treatment

#### ﻿ Selaginella wuyishanensis

Taxon classificationPlantaeSelaginellalesSelaginellaceae

K.W.Xu, X.M.Zhou & Y.F.Duan sp. nov.

BC1869F0-D915-50AB-99C1-BD315040988A

urn:lsid:ipni.org:names:77302501-1

Figs 2 , 3

##### Type.

China. Fujian: Wuyishan City, Mt. Wuyishan, alt. 200m, 27°41'12.82"N, 117°56'12.24"E, 25 Nov. 2021, *Ke-Wang Xu et al. WY21* (holotype: NF!; isotype: PYU!).

##### Diagnosis.

**Figure 3.**
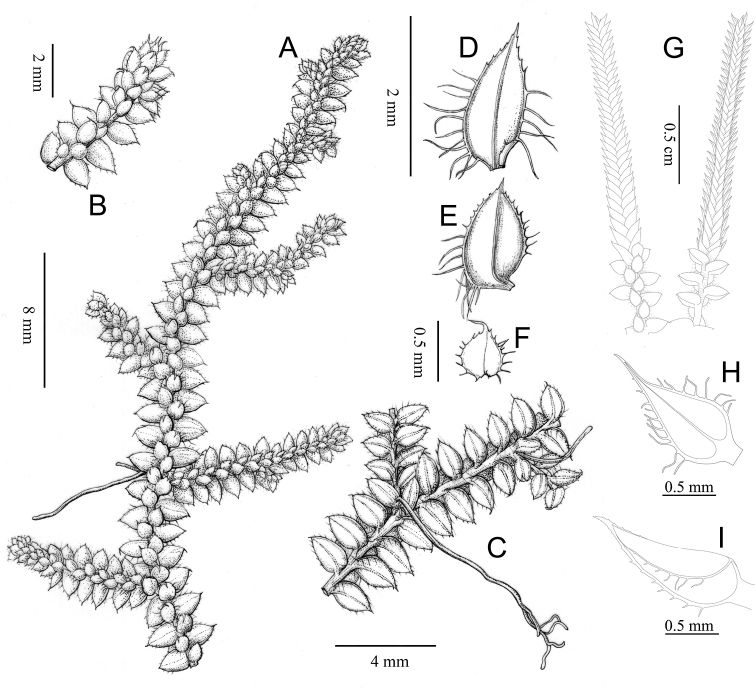
Illustration of *Selaginellawuyishanensis***A** habit **B** adaxial view of branch **C** abaxial view of branch with rhizophore **D** axillary leaf **E** ventral leaf **F** dorsal leaf **G** strobili **H** ventral sporophyll **I** dorsal sporophyll (**A–F** drawn by Sun YB based on the isotype at NF **G–I** drawn by Wei HJ based on the paratype at CSH).

The new species is most similar to *Selaginellaalbociliata* and *S.lutchuensis* in the habit, sterile leaves, and spores. However, *S.wuyishanensis* can be distinguished from the latter two species by its long leaf cilia (up to 0.6 mm), ovate ventral sporophylls, and the smooth perispore surface of the megaspores (Figs 2, 3).

##### Description.

***Plants*** epilithic, evergreen, creeping, without erect or ascending stems. ***Rhizophores*** present at intervals throughout the length of creeping stem and branches, born on ventral side in the axil of main branches, slender, glabrous, 2–5 cm long, 0.1–0.2 mm in diameter; roots usually forked at the apex. ***Stem*** 5–12 cm long, rarely longer than 12 cm, 3–4 mm in width, irregularly and slightly dichotomously branched. ***Leaves*** arranged in four ranks (two dorsal and two ventral). ***Axillary leaves*** present at branching points, oblong-ovate, base slightly cuneate, apex acuminate, 0.8–1.2 × 2.2–2.5 mm, long ciliate along the margin from base to 2/3 of the axillary leaves, cilia up to 0.8 mm long. ***Ventral leaves*** asymmetrical, those on main stem similar to those on branches, imbricate on stem and branch throughout, ovate, 1.5–2.8 × 0.8–1.2 mm, apex slightly acute to acuminate, acroscopic base rounded, basiscopic base slightly cuneate, margin conspicuously white-margins; acroscopic margins ciliate at the base, cilia up to 0.6 mm long, basiscopic margins nearly entire at the base, ciliate or denticulate upward. ***Dorsal leaves*** symmetrical, ovate to oval, 0.8–1.6 × 0.6–0.9 mm, base rounded, apex aristiform, often reflexed, margins conspicuously white-callous, acroscopic margins sparsely ciliate, cilia up to 0.5 mm long, basiscopic margins ciliate, cilia short, no more than 0.1 mm long. ***Strobili*** usually in pairs or rarely three on the branches, terminal, resupinate, 1–2 cm long, megasporangia usually present at basal sporophylls and microsporangia present at upper ones; dorsal sporophylls ovate-lanceolate, base nearly rounded, apex acuminate, 1.2–1.8 × 0.4–0.8 mm, margin conspicuously white-callous, ciliate along the margin of basal part, cilia short; ventral sporophylls membranous, ovate-lanceolate, base rounded, apex caudate, ca. 0.6 × 1.3 mm, ciliate along the margin, cilia ca. 0.3 mm long. ***Megaspores*** yellow, trilete, oblate spheroid to subglobose, equatorial diameter 240–260 μm; perispore reticulate ornamentation with fine muri. ***Microspores*** reddish orange, trilete, hemispheric, equatorial diameter 35–42 μm; verrucate ornamentation of microspore covered with dense rodlets.

##### Distribution and habitat.

*Selaginellawuyishanensis* is known only from Fujian Province, East China. Three populations were observed to grow on rocks of the Danxia landform in evergreen broad-leaved forests at elevations of ca. 200–800 m.

##### Additional specimens examined.

China. Fujian: Wuyishan City, Mt. Wuyishan, alt. 327 m, 27°41'12.82"N, 117°56'12.24"E, 25 Nov. 2021, *Ke-Wang Xu et al. WY521* (NF); the same locality, alt. 280 m, 27°39'17"N, 117°57'50"E, 27 Nov. 2021, *Ke-Wang Xu et al. WY596* (NF); the same locality, *Ke-Wang Xu et al. WY597* (NF); the same locality, *Ke-Wang Xu et al. WY598* (NF); Yongan City, Tianbaoyan National Nature Reserve, 25°57'11"N, 117°33'14"E, 1 Nov. 2020, *Wei &Chen JSL7744A* (CSH).

##### Etymology.

The species epithet is based on the name of the famous mount Wuyishan, referring to the type locality of the new species.

### ﻿Key to *Selaginellawuyishanensis* and its closely related species and morphologically similar species in Fujian Province

**Table:** 

1	Leave margins denticulate and not white-margined	**2**
–	Leave margins more or less ciliate and/or white-margined	**3**
2	Strobili non-resupinate	***S.nipponica***
–	Strobili resupinate	***S.heterostachys***
3	Leaves not white-margined, both sides of ventral leaves long ciliolate at margins	**4**
–	Leaves white-margined, acroscopic base of ventral leaves long ciliolate at margins, elsewhere denticulate or subentire	***S.xipholepis***
4	Ventral leave margins with short cilia up to 0.2 mm; dorsal leave margins loosely serrulate	***S.lutchensis***
–	Ventral and dorsal leave margins with cilia up to 0.6 mm	**5**
5	Ventral sporophylls ovate-lanceolate with length-to-width ratio of ca. 3.2; megaspore surfaces with fine and low papillae structure	***S.albociliata***
–	Ventral sporophylls ovate with length-to-width ratio of ca. 2.4; megaspore surfaces smooth	***S.wuyishanensis***

## Supplementary Material

XML Treatment for Selaginella wuyishanensis

## ﻿Appendix 1

List of taxa sampled with information related to taxonomy, GenBank accession numbers (*rbcL*, 5.8S+ITS2), references, and voucher information. Herbarium acronyms follow Index Herbariorum (Thiers 2018).

***Selaginellaalbociliata* P.S.Wang** (1) *L.-B. Zhang et al. 5302* (CDBI), China (Guangxi), KT161379 (Zhou et al. 2016), KT161648 (Zhou et al. 2016); (2) *X.-C. Zhang 7242* (PE), China (Guizhou), MH814882 (Shalimov et al. 2019), —; (3) *L.-B. Zhang 526* (CDBI), China (Guizhou), ON994457 (this study), —. ***Selaginellaalutacea* Spring***Korall 2006-9* (S), Malaysia: KY022958 (Weststrand and Korall 2016b), —. ***Selaginellaamblyphylla* Alston** (1) *X.-C. Zhang 2924* (PE), China (Yunnan), MH814883 (Shalimov et al. 2019), —; (2) *X.-C. Zhang 7951* (PE), China (Yunnan), MH814884 (Shalimov et al. 2019), —. ***Selaginellaarbuscula* (Kaulf.) Spring** (1) *Schuettpelz 1941* (US), French Polynesia (Marquesas Islands): MT216108 (Steier and Schuettpelz unpublished); —; (2) *Wood 13746* (PTBG), Hawaii (Maui, Kipahulu): KT161388 (Zhou et al. 2016), KT161657 (Zhou et al. 2016). ***Selaginellabanksii* Alston***Grant 3563* (L), French Polynesia: KY022972 (Weststrand and Korall 2016b), —. ***Selaginellabehrmanniana* Hieron.***Johns 8937*, (L), Indonesia: KY022973 (Weststrand and Korall 2016b), —. ***Selaginellabisulcata* Spring***W.-M. Chu et al. 31292* (PYU), China (Yunnan): KT161404 (Zhou et al. 2016), KT161673 (Zhou et al. 2016). ***Selaginellabodinieri* Hieron.** ex Christ (1) *W.-B. Ju & H.-N. Deng HGX13005* (CDBI), China (Sichuan): KT161413 (Zhou et al. 2016), KT161678 (Zhou et al. 2016); (2) *L.-B. Zhang et al. 5177* (CDBI), China (Guangxi): KT161411 (Zhou et al. 2016), KT161679 (Zhou et al. 2016). ***Selaginellaboninensis* Baker***TNS766618* (TNS), Japan (Tokyo): AB574642 (Ebihara et al. 2010), —. ***Selaginellabrachystachya* (Hook. & Grev.) Spring** (1) *J. Klackenberg 434* (S), Sri Lanka: KY022980 (Weststrand and Korall 2016b), —; (2) *W.A. Sledge 913* (L), Sri Lanka: KY022979 (Weststrand and Korall 2016b), —. ***Selaginellachaetoloma* Alston** (1) *Z.-Y. Guo 2016014* (PE), China (Guizhou): MH814888 (Shalimov et al. 2019), —; (2) *X.-C. Zhang 7347* (PE), China (Guizhou): MH814889 (Shalimov et al. 2019), —. ***Selaginellachingii* Alston** (1) *L.-B. Zhang et al. 6587* (CDBI, MO, VNMN, PYU), Vietnam (Lang Son): KT161417 (Zhou et al. 2016), KT161683 (Zhou et al. 2016); (2) *L.-B. Zhang et al. 6594* (CDBI, MO, VNMN, PYU), Vietnam (Lang Son): KT161416 (Zhou et al. 2016), KT161868 (Zhou et al. 2016). ***Selaginellachrysocaulos* (Hook. & Grev.) Spring** (1) *D. E. Boufford et al. 33036* (A), China (Sichuan): KY022955 (Weststrand and Korall 2016b), —; (2) X.-C. Zhang 86 (PE), China (Sichuan): MH814891 (Shalimov et al. 2019), —. ***Selaginellaciliaris* (Retz.) Spring** (1) *BVBRI035-20*, MT795923 (Patel and Reddy 2020), —; (2) *Jiang 310* (PYU, CDBI), China (Hainan): KT161428 (Zhou et al. 2016), KT161691 (Zhou et al. 2016); (3) Unknown (Unknown), Peninsula Malaysia: EU126658 (Yi et al. 2007), —. ***Selaginellacoriaceifolia* X.M.Zhou, N.T.Lu & Li Bing Zhang** (1) *L.-B. Zhang et al. 7307* (CDBI, MO, VNMN), Vietnam (Quang Binh): MT386596 (Ye et al. 2020), MZ570596 (He et al. 2021); (2) L.-B. *Zhang et al. 7371* (CDBI, MO, VNMN), Vietnam (Quang Binh): MT386598 (Ye et al. 2020), MT386595 (Ye et al. 2020). ***Selaginelladecipiens* Warb.** (1) *L.-B. Zhang et al. 6764* (CDBI, MO, VNMN, PYU), Vietnam (Bac Kan): KT161438 (Zhou et al. 2016), KT161698 (Zhou et al. 2016); (2) *L.-B. Zhang et al. 6761* (CDBI, MO, VNMN, PYU), Vietnam (Bac Kan): KT161439 (Zhou et al. 2016), KT161697 (Zhou et al. 2016). ***Selaginelladenticulata* (L.) Spring***Korall & Eriksson 715* (S), Unknown: AJ010853 (Korall et al. 1999), —. ***Selaginelladianzhongensis* X.C.Zhang***Zhu Y.-M. 8158* (PE), China (Yunnan): MH814909 (Shalimov et al. 2019), —. ***Selaginelladrepanophylla* Alston** L.-B. *Zhang et al. 5117* (CDBI), China (Guangxi): KT161447, KT161703 (Zhou et al. 2016); ***Selaginellaeffusa* Alston** (1) *S.-Y. Dong 2470* (PYU), China (Guangdong): KT161453 (Zhou et al. 2016), KT161705 (Zhou et al. 2016); (2) *L.-B. Zhang et al. 5438* (CDBI), China (Guangxi): KT161450 (Zhou et al. 2016), KT161706 (Zhou et al. 2016); (3) *L.-B. Zhang et al. 5442* (CDBI), China (Guangxi): KT161451 (Zhou et al. 2016), KT161707 (Zhou et al. 2016). ***Selaginellagoudotiana* Spring** M. *Thulin and H. Razafindraibe 11750* (UPS), Madagascar: KY023039 (Weststrand and Korall 2016b), —. **Selaginellagoudotianavar.abyssinica (Spring) Bizzarri** R.E.G. *Pichi Sermolli 6756* (L), Ethiopia: KY023038 (Weststrand and Korall 2016b), —. ***Selaginellakanehirae* Alston***Wood 13568* (PTBG), F.S.M. (Caroline Is., Pohnpei): KT161495 (Zhou et al. 2016), KT161745 (Zhou et al. 2016). ***Selaginellakurzii* Baker** (1) *X.-M. Zhou et al. PYU-S-2105* (PYU), China (Yunnan): MZ532022 (He et al. 2021); MZ570598 (He et al. 2021); (2) *X.-C. Zhang 1934* (PE), China (Yunnan): MH814898 (Shalimov et al. 2019), —. ***Selaginellalabordei* Hieron. ex Christ** (1) *X.-F. Gao et al. DJY03894* (CDBI), China (Sichuan): KT161502 (Zhou et al. 2016), KT161750 (Zhou et al. 2016); (2) *H. Smith 2345* (S), China (Sichuan), KY023059 (Weststrand and Korall 2016b), —. ***Selaginellalaxa* Spring** (1) *Schuettpelz 1913* (US), French Polynesia (Marquesas Islands), MT216111 (He et al. 2021); (2) *T. G. Yuncker 15994* (U); Tonga, KY023063, —. ***Selaginellaleptophylla* Baker** (1) *L.-B. Zhang et al. 5199* (CDBI), China (Guangxi): KT161511 (Zhou et al. 2016), KT161758 (Zhou et al. 2016); (2) *X.-M. Zhou & al. DJY05380* (CDBI), China (Sichuan): KT161513 (Zhou et al. 2016), KT161756 (Zhou et al. 2016). ***Selaginellalutchuensis* Koidz.** (1) *TNS101683* (TNS), Japan: MT680176 (Zhang et al. 2021), —; (2) *TNS759343* (TNS), Japan (Okinawa): AB574648 (Ebihara et al. 2010), —. ***Selaginellamegaphylla* Baker** (1) *X.-H. Jin 19301* (PE), China (Xizang): MH814901 (Shalimov et al. 2019), —; (2) *X.-M. Zhou YLZB2185* (CDBI, PYU), China (Xizang): ON994456 (this study), ON994203 (this study). ***Selaginellamenziesii* (Hook. & Grev.) Spring***D. P. Rogers s.n. (Die XI-10-46)* (U), Hawaii, KY023079 (Weststrand and Korall 2016b), —. ***Selaginellaminiatospora* (Dalzell) Bak.** (1) J. *Klackenberg and R. Lundin 567* (S), India (Kerala): KY023081 (Weststrand and Korall 2016b), —; (2) *C. van Hardeveld and H. H. van der Werff 120* (U), India (Tamil Nadu): KY023080 (Weststrand and Korall 2016b), —. ***Selaginellaminutifolia* Spring***Larsen et al. 1389* (S), Thailand: KY023082 (Weststrand and Korall 2016b), —. ***Selaginellamittenii* Baker***van Steenis 24105* (L), South Africa: KY023083 (Weststrand and Korall 2016b), —. ***Selaginellamonospora* Spring** (1) *L.-B. Zhang & al. 6430* (CDBI, MO, VNMN, PYU), Vietnam (Vinh Phuc): KT161537 (Zhou et al. 2016), KT161782 (Zhou et al. 2016); (2) *L. Zhang 1296*, China (Yunnan): MZ532023 (this study), —. ***Selaginellamorganii* Zeiller***P. Korall 2006: 29* (S), Peninsular Malaysia: KY023088 (Weststrand and Korall 2016b), —. ***Selaginellaneocaledonica* Baker** (1) KY985453 (Klaus et al.2016), —; (2) *N. Wikström 244* (S); New Caledonia, KY023095 (Weststrand and Korall 2016b), —. ***Selaginellanipponica* Franch. & Sav.***TNS738139* (TNS), Japan (Tokyo): AB574649 (Ebihara et al. 2010), —. ***Selaginellaornata* (Hook. & Grev.) Spring** (1) *L.-B. Zhang et al. 5200* (CDBI), China (Guangxi): KT161524 (Zhou et al. 2016), KT161770 (Zhou et al. 2016); (2) *W.-M. Chu & al. 8226* (PYU), China (Yunnan): KT161525 (Zhou et al. 2016), KT161767 (Zhou et al. 2016). ***Selaginellaqingchengshanensis* Li Bing Zhang & X.M.Zhou** (1) *X.-F. Gao et al. DJY04053* (CDBI), China (Sichuan): KT161381 (Zhou et al. 2016), KT161649 (Zhou et al. 2016); (2) *Z.-L. Liang & X. Pu 056* (CDBI, PYU), China (Sichuan): MZ532027 (He et al. 2021), MZ570603 (He et al. 2021). ***Selaginellareineckei* Hieron.** (1) *K.R. Wood 16944* (PTBG), Samoa (Savaii): MT657902 (Nitta et al. 2020), —; (2) *H. S. McKee 2907 P7338* (L); Samoa, KY023129 (Weststrand and Korall 2016b), —. ***Selaginellarepanda* (Desv. & Poir.) Spring** (1) *Z.-R. He & X.-M. Zhou 119* (PYU, CDBI), China (Yunnan): KT161583 (Zhou et al. 2016), KT161816 (Zhou et al. 2016); (2) *He & Jiang 405-1* (CDBI), China (Yunnan): KT161584 (Zhou et al. 2016), —. ***Selaginellareticulata* (Hook. & Grev.) Spring***C.R. Fraser-Jenkins 1653* (L), Nepal: KY022956 (Weststrand and Korall 2016b), —. ***Selaginellasubvaginata* X.C.Zhang & Shalimov** (1) *Liu H. 182* (PE) China (Sichuan): MT680177 (Zhang et al. 2020), —; (2) *X.-C. Zhang et al. 9450* (PE) China (Sichuan): MT680181 (Zhang et al. 2020), —. ***Selaginellatrichophylla* K.H.Shing** (1) *L.-B. Zhang et al. 6784* (CDBI, MO, VNMN, PYU), Vietnam (Cao Bang): KT161624 (Zhou et al. 2016), KT161849 (Zhou et al. 2016); (2) *W.-M. Chu & al. 29310* (PYU), China (Yunnan): KT161622 (Zhou et al. 2016), KT161846 (Zhou et al. 2016). ***Selaginellauncinata* (Desv.) Spring***Zhang & Zhou DJY04101* (CDBI), China (Sichuan): KT161626 (Zhou et al. 2016), KT161852 (Zhou et al. 2016). ***Selaginellavaginata* Spring** (1) *X.-M. Zhou 012* (CDBI), China (Sichuan): KT161434 (Zhou et al. 2016); —; (2) *X.-M. Zhou & al. DJY07488* (CDBI), China (Sichuan): KT161432 (Zhou et al. 2016), KT161694 (Zhou et al. 2016). ***Selaginellawhitmeei* Baker***K.R. Wood 17032* (PTBG), Samoa (Savaii), MT657910 (Nitta et al. 2020), —. ***Selaginellawuyishanensis*** (1) *K.-W. Xu WY596* (PYU), China (Fujian): ON994453 (this study), —; (2) *K.-W. Xu WY598* (PYU), China (Fujian): ON994454 (this study), —; (3) *H.-J. Wei JSL7744A* (CSH), China (Fujian): ON994455 (this study), ON994202 (this study). ***Selaginellaxipholepis* Baker** (1) *S.-Y. Dong 2377* (PYU), China (Guangdong): KT161645 (Zhou et al. 2016), —; (2) *L.-B. Zhang & al. 6668* (CDBI, MO, VNMN, PYU), Vietnam (Bac Kan): KT161646 (Zhou et al. 2016), KT161867 (Zhou et al. 2016). ***Selaginellayunckeri* Alston***T. G. Yuncker 15933* (U); Tonga, KY023182 (Weststrand and Korall 2016b), —.
